# Irritability, Negative Life Events, and the Course of Anxiety and Depressive Symptoms in a Clinical Sample of Youth: A Longitudinal Study

**DOI:** 10.1016/j.jaacop.2023.09.001

**Published:** 2023-09-15

**Authors:** Camille Archer, Tatiana Meza-Cervera, Brooke Scheinberg, Katharina Kircanski, Melissa A. Brotman, Daniel S. Pine, Ellen Leibenluft, Julia O. Linke

**Affiliations:** a Emotion and Development Branch, National Institute of Mental Health, National Institutes of Health, Bethesda, Maryland; b Department of Psychology, University of Freiburg, Freiburg im Breisgau, Germany

**Keywords:** anxiety, depression, irritability, negative life events, trajectories

## Abstract

**Objective:**

Irritability, the tendency to react with anger, and the experience of negative life events (NLE) have independently been associated with the emergence of anxiety and depression. This study investigated how irritability and cumulative effects of NLE interactively predict the course of anxiety and depression in the context of common psychiatric disorders.

**Method:**

Study participants were 432 youth with no psychiatric diagnosis or with a diagnosis of an anxiety disorder, attention-deficit/hyperactivity disorder, or disruptive mood dysregulation disorder. At baseline, we assessed NLE, parent and youth reports of irritability and anxiety, and youth reports of depression. Symptoms were annually reassessed for up to 4 years.

**Results:**

In youth without psychiatric diagnoses but with elevated baseline irritability, the presence of NLE predicted decreasing anxiety, while the absence of NLE predicted increasing anxiety. In youth with an anxiety disorder, elevated baseline irritability predicted decreasing anxiety independent of NLE, while a large cumulative effect of NLE predicted increasing depression. NLE predicted persisting mild anxiety in attention-deficit/hyperactivity disorder and persisting mild depressive symptoms in disruptive mood dysregulation disorder.

**Conclusion:**

These findings suggest that, particularly in nonreferred samples, NLE might moderate the relation between irritability and future anxiety such that irritability/anger in the context of NLE can positively affect the course of anxiety. Future work replicating this finding while repeatedly measuring NLE and rigorously controlling for potentially confounding effects of treatment is warranted.

Developmental psychopathology integrates developmental and clinical science to understand the origin and course of psychiatric disorders in youth.^1^^,^^2^ In a clinically referred sample of youth, we applied this framework to examine the interplay of 2 risk factors for anxiety and depression over time: irritability and negative life events (NLE). The developmental psychopathology approach emphasizes the reciprocal and transactional nature of psychobiological vulnerabilities (eg, irritability) and environmental challenges (eg, NLE) in predicting psychiatric outcomes. However, the nature of this interaction might depend on the syndromal context (SC) (ie, the absence or presence of a specific mental disorder) (Figure 1A). To date, no study to our knowledge has investigated whether and how irritability and NLE, in concert, affect the course of anxiety and depressive symptoms across SCs. Such knowledge might guide clinical decision making and allow for targeted early interventions.

**Figure 1:**
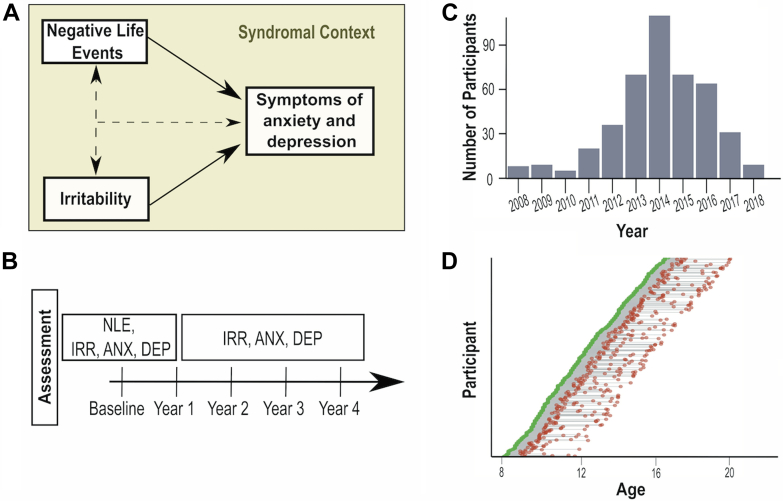
Conceptual Overview and Recruitment ***Note:****(A) Our conceptual framework. Solid lines represent relations supported by previous research, and dashed lines represent hypothesized relations. It shows a transactional relationship between negative life events and irritability and well-established unique as well as hypothesized shared contributions of these 2 variables to anxiety and depressive symptoms. The syndromal context in the background is assumed to affect all established and hypothetical relationships. (B) An overview of the study design. (C) The number of participants we enrolled per year. (D) The age at enrollment and the number of time points completed. Each participant completed the 1-year assessment, and data were missing randomly for the following years. ANX = anxiety; DEP = depression; IRR = irritability; NLE = negative life events.*

Irritability is the tendency to react angrily to slight provocations and disagreements.^3^ In community samples, irritability has been established as a predictor of anxiety and depressive symptoms.^4^, ^5^, ^6^ However, phasic irritability, defined as intense surges of anger in response to frustration,^7^ has also been linked to a decrease in anxiety over time.^8^ These diverging findings might be related to the multifaceted nature of irritability, which is thought to have a tonic (ie, mood) and phasic (ie, outburst) component.^9^^,^^10^ In particular, phasic irritability in response to threats might constitute an active coping strategy that aids individuals in overcoming anxiety.

While the developmental literature views irritability as a high-risk temperament,^11^ it is also studied clinically as an impairing symptom that cuts across diagnostic boundaries.^9^^,^^11^ The findings from the few studies examining the relation between irritability and future anxiety and depression in specific SCs broadly align with findings in community samples. That is, in attention-deficit/hyperactivity disorder (ADHD), irritability has been linked to future depressive symptoms,^12^ and in the context of anxiety disorders, irritability has been linked to a further increase in anxiety.^13^

NLE are broadly defined as unpleasant, uncontrollable, and generally stressful experiences that negatively impact people’s lives.^14^ NLE might have cumulative negative effects in terms of a dose–response relation on mental health.^15^, ^16^, ^17^ Indeed, similar to irritability, NLE have been associated with the emergence of anxiety and depression.^18^, ^19^, ^20^ First evidence also suggests that NLE lead to an increase in anger and irritability.^15^^,^^21^^,^^22^ However, potentially moderating effects of SC have not been studied.

In sum, irritability research and NLE research represent largely independent strands of literature. Models of developmental psychopathology suggest that high irritability may increase sensitivity of youth to environmental influences, such as NLE, leading to the emergence of psychopathology.^23^, ^24^, ^25^, ^26^ Indeed, a recent study showed that stressful life events moderate the association between temperamental negative emotionality, which encompasses irritability, and the emergence of internalizing symptoms.^27^ However, we are unaware of studies investigating how irritability and NLE interactively influence future anxiety or depression across SCs. To close this gap, we examined how irritability and NLE interactively affect the course of anxiety and depressive symptoms across 4 SCs: the absence of psychopathology, ADHD or an anxiety disorder where irritability is common,^28^^,^^29^ or disruptive mood dysregulation disorder (DMDD) where irritability is the core symptom. Further, we explored whether NLE influence the course of irritability across these 4 SCs (Figure 1A). Although prior work suggests that SC might moderate the interactive effects of irritability and NLE,^15^ the empirical basis did not allow for the formulation of specific hypotheses.

## Method

### Participants

We recruited 432 youth (47.7% identifying as male) as part of ongoing clinical studies seeking to identify the neurobiological underpinnings of pediatric anxiety and irritability. Some participants, particularly youth with an anxiety disorder, were seeking treatment, whereas others had established relationships with health care providers in the community. The mean [SD] age when entering the study was 13.0 [2.74] years (range 8.1-18.1 years). Of participants, 13.9% identified as African American, 2.8% identified as Asian, 68.5% identified as White, and 10.7% reported multiple racial identities. Further, 11% identified as Latinx or Hispanic. Annual household income was less than $90,000 in 21% of participating families, between $90,000 and $180,000 in 41%, and greater than $180,000 in 26%. Exclusion criteria included IQ below 70 (mean [SD] = 112 [13.1]) as determined with the Wechsler Abbreviated Scale of Intelligence^30^ and diagnoses of neurological, autism spectrum, posttraumatic stress, psychotic, bipolar, or substance use disorders. All procedures were approved by the Institutional Review Board, and written informed consent and assent were obtained.

At enrollment, a master- or doctoral-level clinician determined diagnostic status of youth using the Schedule for Affective Disorders and Schizophrenia for School-Age Children–Present and Lifetime Version (K-SADS-PL).^31^ At baseline, the sample comprised 219 youth without a psychiatric diagnosis and 213 youth with at least one of the following disorders: ADHD (n = 75), an anxiety disorder (n = 145), and DMDD (n = 64). A primary diagnosis of ADHD or DMDD was more common among youth identifying as male. Among youth with a DMDD diagnosis, fewer participants identified as African American, and, on average, this group had slightly lower intelligence scores (see Table S1, available online). At enrollment, youth without a psychiatric diagnosis or with a primary anxiety disorder diagnosis did not take any psychotropic medications. Of the participants with a primary diagnosis of ADHD or DMDD, 68 youth took psychotropic medications (n = 29 antidepressants, n = 19 antipsychotics, n = 9 anticonvulsants, n = 22 nonstimulant treatments for ADHD, n = 45 stimulants). On average, youth with a DMDD diagnosis were taking more psychotropic medication than youth with an ADHD diagnosis (see Table S1, available online).

### Study Design

Participants were recruited on a rolling basis (Figure 1B) between 2008 and 2018 (Figure 1C). After study eligibility was confirmed, information regarding the cumulative effect of NLE up to the time of recruitment and current symptoms of irritability, anxiety, and depressive symptoms was obtained. After the initial assessment, these symptoms were rated yearly for up to 4 years (Figure 1D). As parents and youth often diverge in their assessment of irritability^32^ and anxiety,^33^ and accumulating evidence suggests that both perspectives are meaningful and discrepancies should not be disregarded as noise,^34^ we did not average symptom ratings across informants, but rather analyzed them independently.

#### Negative Life Events

NLE were assessed with the Life Events Checklist, a 46-item self-report questionnaire.^35^ Youth indicated whether they experienced an event from a list at any point in their life and rated the valence of the event (positive vs negative) and its effect on a 4-point Likert scale ranging from 1 (no effect) to 4 (great effect). The effects of the negative events were summed to reflect their cumulative effect. However, as the resulting distribution was highly left skewed, we recoded the sum scores into an ordinal variable, which was used in our analyses (no NLE; low cumulative NLE effect, score 1-3; Moderate cumulative NLE effect: score 4-7; high cumulative NLE effect: score >7).

#### Youth Irritability

Irritability was rated separately by parents (p-IRR) and youth (y-IRR) using the Affective Reactivity Index (ARI),^36^ which assesses a child’s angry mood and temper outbursts over the past 6 months. The ARI consists of 6 items on a 3-point Likert scale ranging from 0 (not true) to 2 (certainly true), giving a range of possible scores from 0 to 12. The p-IRR and y-IRR ARI ratings show good internal consistency and test-retest reliability (parent-report: α = .92, intraclass correlation coefficient [ICC] = 0.85; youth-report: α = .88, ICC = 0.78).^32^

#### Youth Anxiety

Anxiety was rated separately by parents (p-ANX) and youth (y-ANX) using the Screen for Child Anxiety Related Disorders (SCARED).^37^ The SCARED consists of 41 items rated on a 3-point Likert scale ranging from 0 (not true or hardly ever true) to 2 (very true or often true). Here we analyzed the sum score of all items, which ranges from 0 to 82. The SCARED has good internal consistency and test-retest reliability (parent-report: α = .90, ICC = 0.74-0.86; youth-report: α = .90, ICC = 0.59-0.6).^37^^,^^38^

#### Depressive Symptoms

Youth rated their depressive symptoms using the Children’s Depression Inventory (CDI). The CDI comprises 27 items rated on a 3-point scale increasing in severity from 0 to 2, which are summed to a total score ranging from 0 to 54.^39^ The CDI has moderate to good test-retest reliability (*r*s = 0.50-0.84) and acceptable internal consistency (psychiatric populations: α = .80).^40^

#### Syndromal Context

We coded SC as one factor with 4 conditions: absence of a psychiatric disorder (n = 219), which served as a reference condition, or primary diagnosis of anxiety disorder, ADHD, or DMDD. Youth in the anxiety disorder category (n = 112) did not have comorbid ADHD or DMDD. However, 30% of youth in the ADHD category and 27% of youth in the DMDD category also met the criteria for at least one anxiety disorder. Further, 31% of youth in the DMDD category also fulfilled ADHD criteria. In case of a significant effect, we conducted post hoc analyses testing the robustness of the findings through inclusion or exclusion of comorbid cases.

### Data Analysis

We used multilevel/linear mixed effects models to determine how cumulative effects of NLE and youth- or parent-rated baseline irritability (yB-IRR, pB-IRR) interactively affect the course of y-ANX and p-ANX, and youth-rated depression (DEP). We also explored how cumulative effects of NLE influence the course of y-IRR and p-IRR. Further, we tested whether model performance improved when SC was added to the model.

Analyses were conducted in R (R Foundation for Statistical Computing, Vienna, Austria) using the lmer function from the lme4 package.^41^ We employed a model-building procedure to determine whether adding the parameters of interest (NLE × irritability, SC) improved the model fit. All *p* values were obtained by likelihood ratio tests of the full model with the effect in question against the model without the effect in question. We report marginal *R*^2^ representing the variance explained by the fixed effects and conditional *R*^2^, which is interpreted as the variance explained by the full model.^42^ Residual plots were visually inspected to ensure no apparent deviations from homoscedasticity or normality.

The baseline model described how the outcome measures changed over time:

Level 1: y-ANX_ti_ or p-ANX_ti_ or DEP_ti_ or p-IRR_ti_ or y-IRR_ti_ = β_0i_ + β_1i_(time_ti_) + e_ti_

Level 2: β_0i_ = y_00_ + u_0i_

β_1i_ = y_10_ + u_1i_

Next, we determined the effects of cumulative effects of NLE and either pB-IRR (model 2a) or yB-IRR (model 2b) on the course of the y-ANX, p-ANX, or DEP:

Level 1: y-ANX_ti_ or p-ANX_ti_ or DEP_ti_ = β_0i_ + β_1i_(time_ti_) + e_ti_

Level 2: β_0i_ = y_00_ + y_01_(NLE_i_) + y_02_(pB-IRR_i_ or yB-IRR_i_) + y_03_(NLE_i_ × pB-IRR_i_ or yB-IRR_i_) + u_0i_

β_1i_ = y_10_ + y_11_(NLE_i_) + y_12_(pB-IRR_i_ or yB-IRR_i_) + y_13_(NLE_i_ × pB-IRR_i_ or yB-IRR_i_) + u_1i_

Model 2c examined the effect of NLE on p-IRR and y-IRR:

Level 1: p-IRR_ti_ or y-IRR_ti_ = β_0i_ + β_1i_(time_ti_) + e_ti_

Level 2: β_0i_ = y_00_ + y_01_(NLE_i_) + u_0i_

β_1i_ = y_10_ + y_11_(NLE_i_) + u_1i_

Last, we determined whether the longitudinal effects of cumulative NLE and either pB-IRR (model 3a) or yB-IRR (model 3b) depend on the SC:

Level 1: y-ANX_ti_ or p-ANX_ti_ or DEP_ti_ = β_0i_ + β_1i_(time_ti_) + e_ti_

Level 2: β_0i_ = y_00_ + y_01_ (NLE_i_) + y_02_(pB-IRR_i_ or yB-IRR_i_) + y_03_(SC_i_) + y_04_(NLE_i_ × pB-IRR_i_ or yB-IRR_i_) + y_05_(NLE_i_ × SC_i_) + y_06_(pB-IRR_i_ or yB-IRR_i_ × SC_i_) + y_07_(NLE_i_ × pB-IRR_i_ or yB-IRR_i_ × SC_i_) + u_0i_

β_1i_ = y_10_ + y_11_ (NLE_i_) + y_12_(pB-IRR_i_ or yB-IRR_i_) + y_13_(SC_i_) + y_14_(NLE_i_ × pB-IRR_i_ or yB-IRR_i_) + y_15_(NLE_i_ × SC_i_) + y_16_(pB-IRR_i_ or yB-IRR_i_ × SC_i_) + y_17_(NLE_i_ × pB-IRR_i_ or yB-IRR_i_ × SC_i_) + u_0i_

Model 3c tested whether the effect of NLE on the course of p-IRR or y-IRR depends on the SC:

Level 1: p-IRR_ti_ or y-IRR_ti_ = β_0i_ + β_1i_(time_ti_) + e_ti_

Level 2: β_0i_ = y_00_ + y_01_(NLE_i_) + y_02_(SC_i_) + y_03_(NLE_i_ × SC_i_) + u_0i_

β_1i_ = y_10_ + y_11_(NLE_i_) + y_12_(SC_i_) + y_13_(NLE_i_ × SC_i_) + u_1i_

Age at enrollment was included as a covariate in all models. We report the final model in “Results.” Model comparisons are shown in Tables S3-S12, available online. We also plotted the raw data as a function of cumulative NLE effects and B-IRR. Given the baseline differences in NLE and B-IRR, the cutoffs used in these graphs vary by SC (cutoffs for high NLE: healthy volunteer ≥2, anxiety disorder, ADHD, DMDD ≥3; cutoffs for high pB-IRR: healthy volunteer ≥1, anxiety disorder ≥4, ADHD ≥6, DMDD ≥8). We also explored whether age altered trajectories of anxiety, depressive symptoms, or irritability. These analyses yielded no significant findings and can be found in the Supplement, available online.

## Results

### Baseline

Across SC, cumulative effects of NLE were relatively low (no NLE, n = 105; low cumulative NLE effect, n = 140; moderate cumulative NLE effect, n = 101; high cumulative NLE effect, n = 86). Cumulative effects of NLE were higher in participants with African American ancestry (*W* = 9259.5, *p* = .033) or an annual household income below $90,000 (*W* = 12923, *p* = .009). This finding is in line with previous work^43^ and highlights the need for future studies in these at-risk populations. NLE were not associated with age, sex, or other racial or ethnic identities (all *p*s > .50).

Youth with no psychiatric disorder reported low levels of irritability, anxiety, and depressive symptoms. Among youth diagnosed with ADHD, irritability, anxiety, and depressive symptoms were mild. Youth with an anxiety disorder diagnosis reported clinically relevant anxiety, mild irritability, and mild depressive symptoms. Youth with a DMDD diagnosis showed moderate irritability paired with mild anxiety and depressive symptoms. For details, see Table S2, available online.

### Retention

We obtained symptom ratings for the complete sample after 1 year. However, attrition was relatively high in years 2 to 4. In the second year, 46% of the original sample were available for assessment (ADHD, 41%; anxiety disorder, 45%; DMDD, 46%; healthy volunteer, 39%). In the third year, symptom ratings from 43% of the original sample were obtained (ADHD, 39%; anxiety disorder, 43%; DMDD, 38%; healthy volunteer, 39%). For the last assessment, we had data from 37% of the original sample (ADHD, 34%; anxiety disorder, 37%; DMDD, 34%; healthy volunteer, 30%). Little’s missing completely at random test indicated no relation between the missingness and any observed or missing values in the symptom measures (χ^2^_1663_ = 1718.67, *p* = .167). Further, missingness was unrelated to age (χ^2^_4_ = 0.99, *p* = .911); gender (χ^2^_4_ = 1.63, *p* = .802); income (χ^2^_8_ = 3.64, *p* = .888); ADHD (χ^2^_4_ = 1.28, *p* = .865), anxiety disorder (χ^2^_4_ = 4.37, *p* = .358), or DMDD (χ^2^_4_ = 2.82, *p* = .588) diagnosis; absence of a psychiatric diagnosis (χ^2^_4_ = 4.82, *p* = .306); severity of irritability (parent: χ^2^_4_ = 1.88, *p* = .759; youth: χ^2^_4_ = 1.16, *p* = .885); anxiety (parent: χ^2^_4_ =1.19, *p* = .880; youth: χ^2^_4_ = 5.82, *p* = .212) or depressive (χ^2^_4_ = 2.12, *p* = .714) symptoms at baseline; or NLE (χ^2^_12_ =8.71, *p* = .727).

### NLE, Irritability, SC, and Course of Anxiety Symptoms

**Figure 2:**
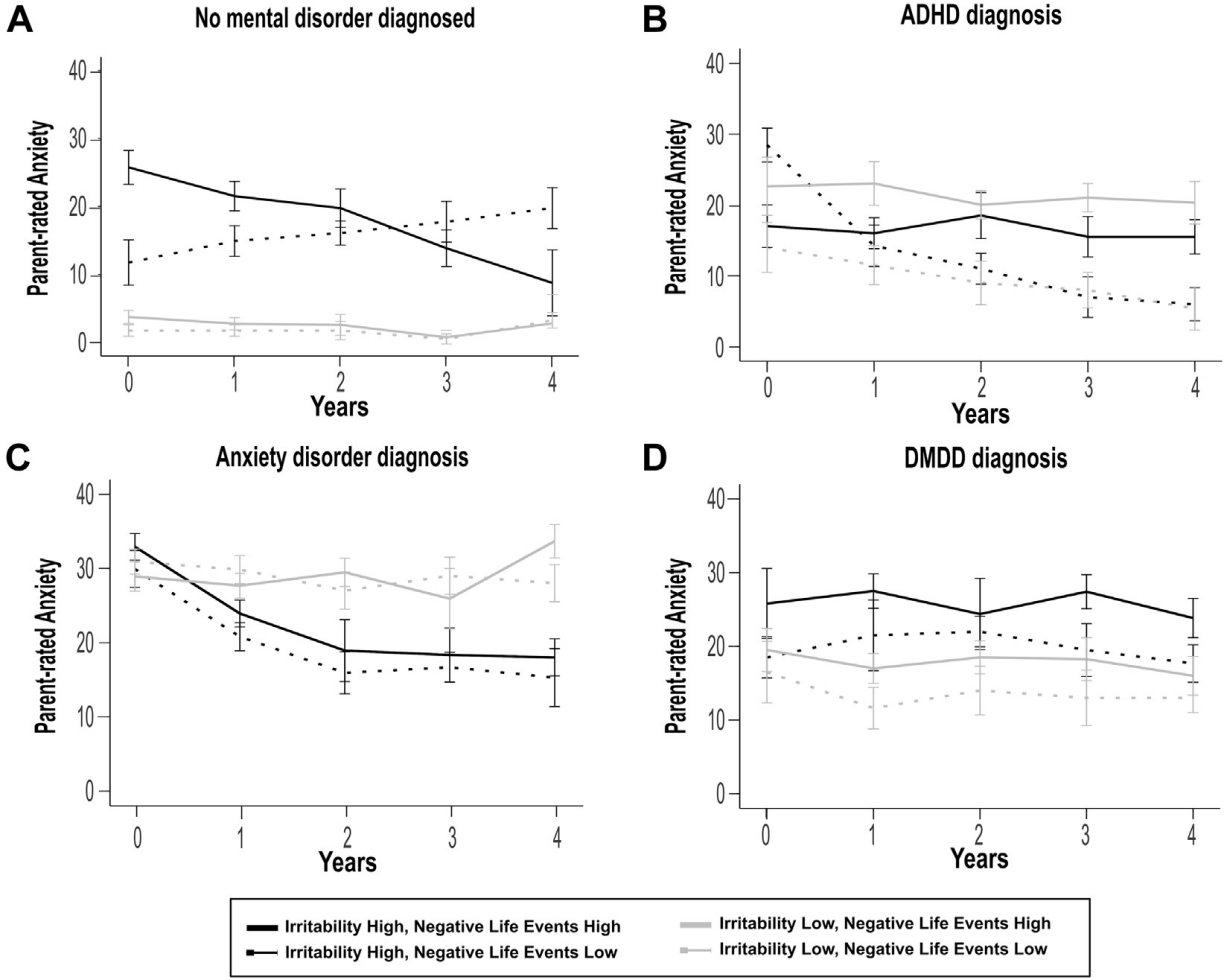
Course of Parent-Rated Anxiety as a Function of Parent-Rated Irritability, Cumulative Adverse Life Events, and Diagnostic Status at Baseline ***Note:****The number of participants in the depicted subgroups varied across syndromal contexts (high irritability, high NLE: n*_*HV*_*= 38, n*_*ANX*_*= 29, n*_*ADHD*_*= 11, n*_*DMDD*_*= 16; high irritability, low NLE: n*_*HV*_*= 47, n*_*ANX*_*= 29, n*_*ADHD*_*= 14, n*_*DMDD*_*= 14; low irritability, high NLE: n*_*HV*_*= 66, n*_*ANX*_*= 52, n*_*ADHD*_*= 15, n*_*DMDD*_*= 17; low irritability, low NLE: n*_*HV*_*= 58, n*_*ANX*_*= 39, n*_*ADHD*_*= 15, n*_*DMDD*_*= 17). ADHD = attention-deficit/hyperactivity disorder; ANX = anxiety; DMDD = disruptive mood dysregulation disorder; HV = healthy volunteer; NLE = negative life events.*

Across participants, y-ANX (β = −1.43, χ^2^_2_ = 36.69, *p* < .001, marginal *R*^2^ = 0.01, conditional *R*^2^ = .74) and p-ANX (β = −.92, χ^2^_2_ = 27.84, *p* < .001, marginal *R*^2^ = 0.03, conditional *R*^2^ = 0.79) decreased over time. However, the course of y-ANX (χ^2^_24_ = 76.75, *p* < .001, marginal *R*^2^ = 0.39, conditional *R*^2^ = 0.80) (Tables S3 and S4, available online) and p-ANX (χ^2^_24_ = 178.82, *p* < .001, marginal *R*^2^ = 0.39, conditional R^2^ = 0.80) (Tables S5 and S6, available online) was best understood as a function of cumulative effects of NLE, B-IRR (yB-IRR for y-ANX, pB-IRR for p-ANX), and SC. More specifically, in the absence of a psychiatric disorder diagnosis, y-ANX and p-ANX remained stable in youth with low B-IRR; decreased in youth with high B-IRR and strong cumulative effects of NLE; and increased in youth with high B-IRR, but no NLE. In the context of anxiety disorders, we saw a decrease in y-ANX and p-ANX in youth with high B-IRR independent of NLE (Figure 2; Figure S1, available online). Results remained when participants with comorbid anxiety disorder and ADHD or DMDD were included in the anxiety disorder category. Results for the subgroup with no psychiatric disorder diagnosis, when using a cutoff of ≥2 to define high irritability, are shown in Figure S2, available online.

### NLE, Irritability, SC, and Course of Depressive Symptoms

**Figure 3:**
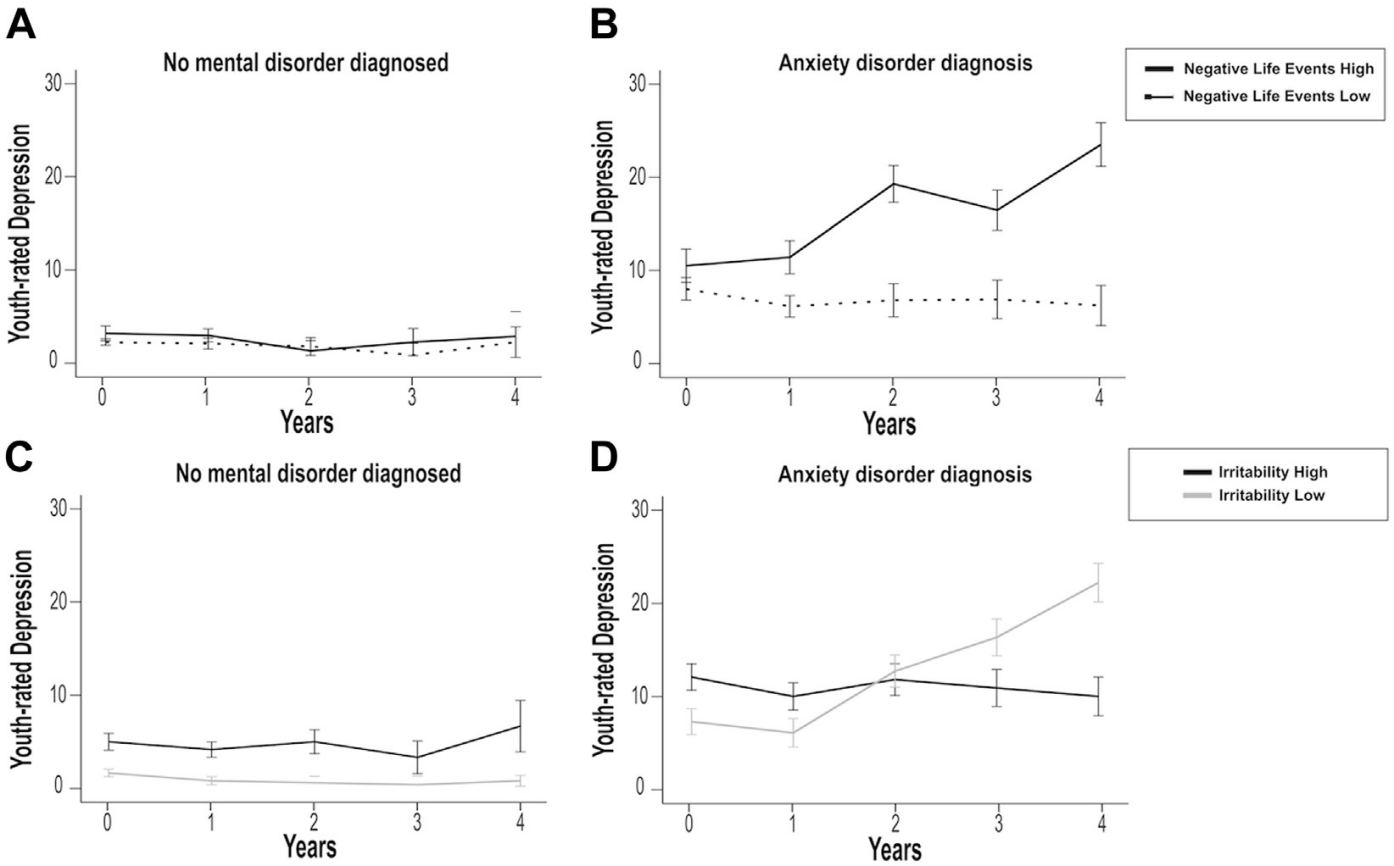
Course of Youth-Rated Depressive Symptoms as a Function of Baseline Irritability and Adverse Life Events in the Context of No Psychiatric Disorder vs Anxiety Disorders ***Note:****The number of participants in the depicted subgroups varied across syndromal contexts (high NLE: n*_*HV*_*= 83, n*_*ANX*_*= 48; low NLE: n*_*HV*_*= 86, n*_*ANX*_*= 49). ANX = anxiety; HV = healthy volunteer; NLE = negative life events.*

Across participants, depressive symptoms remained stable (β = −.24, χ^2^_3_ = 5.68, *p* = .13). However, we observed informant-independent time × NLE × ANX interactions (model 2a: χ^2^_11_ = 66.05, *p* < .001, marginal *R*^2^ = 0.25, conditional *R*^2^ = 0.68; model 2b: χ^2^_11_ = 53.35, *p* < .001, marginal *R*^2^ = 0.37, conditional *R*^2^ = 0.68) (Figure 3; Tables S7 and S8, available online). These interactions showed that in the absence of a psychiatric disorder diagnosis, low-level depressive symptoms remained stable, whereas anxious youth with many NLE developed more depressive symptoms over time. Further, we also saw that low yB-IRR predicted an increase in depressive symptoms (Figure 3). Results remained when participants with comorbid anxiety disorder and ADHD or DMDD were included in the anxiety disorder category.

### NLE, SC, and Course of Irritability

**Figure 4:**
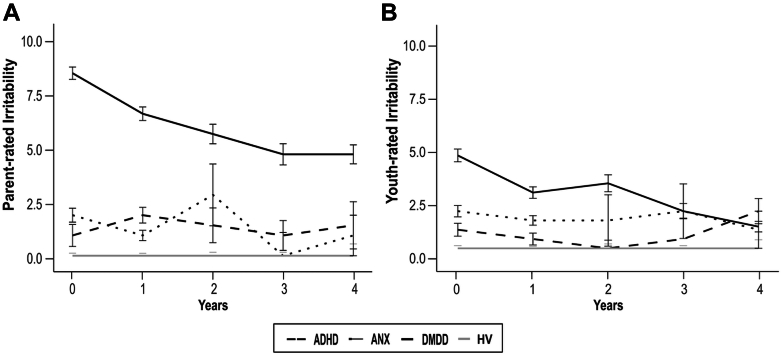
Trajectories of Parent-Rated (A) and Youth-Rated (B) Irritability Across 4 Syndromal Contexts ***Note:****ADHD = attention-deficit/hyperactivity disorder; ANX = anxiety; DMDD = disruptive mood dysregulation disorder; HV = healthy volunteer.*

Independent of the informant, baseline irritability was lower in older youth (yB-IRR: β = −.10; pB-IRR: β = −.14) and higher in youth reporting more NLE (yB-IRR: β = .36) or having a DMDD diagnosis (yB-IRR: β = 3.06; pB-IRR: β = 5.32). Of all predictors, DMDD diagnosis alone influenced the course of irritability (y-IRR: β = −.49, χ^2^_6_ = 117.51, *p* < .001, marginal *R*^2^ = 0.26, conditional *R*^2^ = 0.67; p-IRR: β = −.45, χ^2^_6_ = 236.92, *p* < .001, marginal *R*^2^ = 0.49, conditional *R*^2^ = 0.80) (see Tables S9 and S10 for y-IRR and Tables S11 and S12 for p-IRR, available online). In more detail, y-IRR and p-IRR remained stable at a low level in the absence of a psychiatric disorder and at a moderate level in the context of ADHD and anxiety disorder. However, y-IRR and p-IRR decreased in the context of DMDD (Figure 4). These effects remained when youth with comorbid anxiety disorder or ADHD were excluded from the DMDD category.

## Discussion

The present study examines how irritability and the cumulative effect of NLE affect the course of anxiety and depressive symptoms across SCs. Extending previous findings, we show that in the absence of a psychiatric disorder, the effect of high baseline irritability on the course of anxiety depends on the cumulative effect of NLE. Moreover, in youth with an anxiety disorder, high irritability may be associated with a decrease in anxiety symptoms, while low irritability in the face of impactful NLE relates to an increase in depressive symptoms. There was no support for the hypothesis that the cumulative effect of NLE determines the course of irritability. Irritability remained relatively stable in the context of ADHD, anxiety disorder, and the absence of a psychiatric disorder, but decreased in the context of DMDD, where it was highest at baseline.

Our findings extend previous reports^4^, ^5^, ^6^ that establish irritability as a prospective predictor of anxiety in community samples. These prior studies focused on clinician ratings of irritability,^4^ which tend to correlate strongly with parent reports of irritability,^6^ and did not account for NLE. Consistent with the main finding of these prior studies, we observed an increase in anxiety in our subsample of healthy volunteers, who had high baseline irritability but no NLE. However, we extend prior work by demonstrating that in the context of many NLE, high baseline irritability predicts a decrease in anxiety over time in youth with no psychiatric diagnosis.

Irritability can be viewed as temperament or a symptom, but commonly used irritability measures (eg, ARI) do not differentiate these 2 aspects. Nevertheless, we propose that the observed NLE by irritability interaction may reflect differences between irritability as a temperament disposition and irritability as a symptom/reaction triggered by cumulative effects of NLE. It seems plausible that in the absence of a psychiatric diagnosis and NLE, irritability ratings reflect the temperament/trait known to predict the emergence of anxiety.^44^ However, in the context of many cumulative NLE, irritability, particularly irritable angry behavior, might be understood as a defensive reaction.^45^ Of the 3 defensive modes (ie, fight, flight, and freeze), anxiety has been related to continued flight or freeze responses to stressors/threats such as NLE.^46^^,^^47^ In contrast, active coping responses might foster resilience against anxiety.^48^ Indeed, irritable behavior, defined as intense surges of anger in response to frustration,^7^ has been previously shown to predict a decrease in worries over 3 years in a large community sample of adolescent girls.^8^

In our anxious participants, where disorder-specific stimuli present a constant threat, elevated baseline irritability was also associated with a steeper decrease in anxiety over time. As 4 of the 6 items of our irritability measure focus on phasic irritability, this further supports the notion that irritability in the sense of active coping or fight response might have long-term positive effects on the course of anxiety. Consistent with this conceptualization, we observed an increase in depressive symptoms in anxious youth with low youth-reported baseline irritability. The literature on irritable/angry/aggressive behavior and depression is sparse and somewhat fragmented,^49^ but the failure to execute a fight reaction (ie, arrested anger) in response to threats has been previously linked to depressive symptoms.^50^^,^^51^ However, the causal nature of relations among these variables remains unknown. Future investigations into phasic irritability as a fight response might further our understanding of these complex relations and suggest novel interventions, particularly in anxious youth. However, future work should also explore alternative explanations such that irritability in the context of many cumulative NLE triggers specific behavior in caregivers (eg, more consistent and predictable parenting behavior) that might reduce anxiety.

In the context of ADHD or DMDD, we find no evidence that cumulative effects of NLE or baseline irritability, which are generally elevated in these 2 disorders,^52^ influenced the course of anxiety or depressive symptoms. This might not be too surprising, given recent work that links tonic irritability (ie, grouchy, angry mood) to the emergence of anxiety and depressive symptoms in youth.^8^^,^^53^ Further, scores from the ARI questionnaire likely represent more of the phasic component, as the majority of the items asks for outbursts, and endorsement of the 2 angry mood items is notoriously low. While this supports the focus of our discussion on phasic irritability, we also note that it prevented us from testing the potentially opposing effects of these 2 facets of irritability on the course of anxiety and depressive symptoms. To address this, future work should aim to conceptualize and assess irritability as a multifaceted rather than a unitary construct, either honing into data-driven divisions of existing measures^10^^,^^53^ or using new measures specifically targeting temper outbursts^54^ or angry mood.

Finally, we emphasize that across SCs, we found no evidence that cumulative effects of NLE alter the course of irritability. While irritability decreased over time in youth diagnosed with DMDD, it remained elevated in youth with an anxiety disorder diagnosis or ADHD compared to youth with no psychiatric disorder. Although treatment history was not assessed, given the severity of DMDD, it is highly likely that youth diagnosed with DMDD were treated for this condition. The fact that irritability remained elevated suggests that more work examining efficacious interventions for irritability is needed.

Findings must be interpreted in the light of some limitations. First, our study sample was rather affluent, predominantly White, and restricted to specific diagnostic categories. Thus, replication in other samples, particularly samples with a more diverse socioeconomic and cultural background that also include other diagnostic categories, such as major depressive disorder, is warranted. Second, prevalence of NLE was low, and the retrospective one-time assessment of the cumulative effects of NLE did not allow for the investigation of transactional effects between NLE and symptoms. Additionally, future work should aim to account for NLE that may occur between follow-up assessments, which could influence symptom trajectories. Third, while all participants completed the 1-year follow-up, retention was low for the second, third, and fourth time points. Fourth, youth with a psychiatric disorder diagnosis were not treatment naïve and might have changed their treatment during the study. We did not systematically assess treatment history and treatment change and so could not test whether these variables contribute to the reported effects. Although it appears unlikely that diverse interventions (ie, psychopharmacological interventions, psychological interventions, and combinations) at varying time points known to elicit a variable treatment response^55^, ^56^, ^57^ caused the reported effects, future work assessing the effects of pharmacological and psychosocial interventions on the reported longitudinal relations, is warranted. Fifth, NLE were more prevalent in youth with relatively low household income. We did not control for income, as information was missing in 20% of participants, but future work should aim to study this effect in more detail.

The present study highlights the need to routinely assess irritability as a multifaceted construct (ie, temperament vs symptom, phasic vs tonic) when examining longitudinal relations with anxiety and depressive symptoms. Future work may address this by creating more specific questionnaires that distinguish between irritability as temperament or symptom and between phasic and tonic irritability. Our findings indicate that irritability in the sense of a defensive response to threats such as NLE might relate to resilience against anxiety and depressive symptoms, particularly in the context of an anxiety disorder and the absence of a psychiatric diagnosis.
