# Supplementary material for: Irritability, Negative Life Events, and the Course of Anxiety and Depressive Symptoms in a Clinical Sample of Youth: A Longitudinal Study
Source: JAACAP Open. 2023 Sep 15;2(1):45–54. doi: 10.1016/j.jaacop.2023.09.001 (PMC11062628; doi:10.1016/j.jaacop.2023.09.001)
Supplement: Supplemental Data [file mmc1.docx]

**Supplementary**

**Materials**

**Irritability, Negative Life Events and the course of anxiety and depressive symptoms in a clinical sample of youth: A longitudinal study**

**Table S1. Demographics and clinical data, broken down by primary psychiatric diagnosis.**

|  | **HV**  **(n = 219)** | **ANX**  **(n = 104)** | **ADHD (n = 45)** | **DMDD (n = 64)** | **Test-statistic** | **p-value** |
| --- | --- | --- | --- | --- | --- | --- |
| **Demographics** |  |  |  |  |  |  |
| **Gender**, percent identifying as male | 46 % | 42 % | 60 % | 59 % | Chi^2^_(3)_ = 7.90 | ***.049*** |
| **Age**, mean (SD) | 13.2 (2.69) | 12.6 (2.85) | 12.2 (2.61) | 13.1 (2.69) | F_(1,430)_ = 1.08 | .299 |
| **Race and Ethnicity** |  |  |  |  |  |  |
| Asian, percent **^a^** | 2 % | 3 % | 3 % | 2 % |  | .829 |
| African American, percent **^a^** | 18 % | 11 % | 17 % | 2 % |  | ***.001*** |
| White, percent | 64 % | 70 % | 66 % | 81% | Chi^2^_(3)_ = 6.82 | .078 |
| Multiple, percent **^a^** | 10 % | 12 % | 11 % | 11 % |  | .880 |
| Latinx or Hispanic, percent **^a^** | 12 % | 12 % | 11 % | 8 % |  | .810 |
| **Annual Household Income** |  |  |  |  |  |  |
| < $90.000, percent | 21 % | 16 % | 20 % | 31 % | Chi^2^_(3)_ = 5.88 | .117 |
| $90.000 – $180.000, percent | 41 % | 46 % | 37 % | 36 % | Chi^2^_(3)_ = 2.32 | .510 |
| > $180.000, percent | 23 % | 34 % | 29 % | 19 % | Chi^2^_(3)_ = 7.04 | .071 |
| **Clinical variables** |  |  |  |  |  |  |
| **Psychiatric diagnoses** |  |  |  |  |  |  |
| ANX, percent **^b^** | 0 % | 100 % | 30 % | 27 % |  |  |
| ADHD, percent **^b^** | 0 % | 0 % | 100 % | 31 % |  |  |
| DMDD, percent **^b^** | 0 % | 0 % | 0 % | 100 % |  |  |
| **Psychotropic medication** |  |  |  |  |  |  |
| No medication | 100 % | 100 % | 56 % | 28 % | Chi^2^_(3)_ = 218.74 | ***<.001*** |
| Antidepressants **^a^** | 0 % | 0 % | 5 % | 34 % |  | .053 |
| Antipsychotics **^a^** | 0 % | 0 % | 0 % | 28 % |  | ***.001*** |
| Non-Stimulant ADHD medication **^a^** | 0 % | 0 % | 37 % | 50 % |  | ***<.001*** |
| Stimulant ADHD medication **^a^** | 0 % | 0 % | 11 % | 27 % |  | .071 |
| **IQ** | 113 (12.5) | 113 (13.2) | 113 (15.7) | 108 (12.8) | F_(1,415)_ = 5.14 | ***.024*** |

**^Abbreviations: ADHD,^** ^Attention-Deficit/ Hyperactivity Disorder;^ **^ANX,^** ^anxiety disorder;^ **^DMDD,^** ^Disruptive Mood Dysregulation Disorder;^ **^HV,^** ^Healthy volunteer;^ **^IQ,^** ^intelligence quotient;^ **^SD,^** ^standard deviation^

**^a^** ^As cell sizes were small, we used Fisher’s Exact test, which does not produce a test statistic.^

**^b^** ^Groups with certain primary psychiatric disorder diagnoses are expected to differ in these variables.^

**Supplement 1: Exploratory analyses of developmental effects.**

We also explored whether the course of anxiety, depressive symptoms, and irritability varied between youth enrolled during late childhood (<= 12 years), early (13-15 years), and late adolescence (16-18 years). Results of these analyses did not indicate age-dependent trajectories of parent-rated irritability (β_timexage_=−.02, p = .624, Chi^2^_(1)_=0.37, p=.542, marginal R^2^=.02, conditional R^2^=.78), youth-rated irritability (β_timexage_=−.04, p = .374, Chi^2^_(1)_=0.86, p=.352, marginal R^2^=.04, conditional R^2^=.65), parent-rated anxiety (β_timexage_=.35, p = .164, Chi^2^_(1)_=1.40, p=.236, marginal R^2^=.03, conditional R^2^=.79) or youth-rated anxiety (β_timexage_=.27, p = .323, Chi^2^_(1)_=1.29, p=.256, marginal R^2^=.01, conditional R^2^=.74). For youth-rated depressive symptoms, we saw a marginal effect (β_timexage_=.38, p = .063, Chi^2^_(1)_=3.46, p=.063, marginal R^2^=.00, conditional R^2^=.64).

**Table S2. Overview of dependent and independent variables at baseline.**

| **Syndromal Context** | **Mean** | **SD** | **Min** | **Max** | **Skewness** |
| --- | --- | --- | --- | --- | --- |
|  | **NLE** | | | | |
| ***HV*** | 3.5 | 5.99 | 0 | 69 | 6.7 |
| ***ANX*** | 6.2 | 9.14 | 0 | 83 | 5.2 |
| ***ADHD*** | 5.2 | 6.23 | 0 | 24 | 1.6 |
| ***DMDD*** | 5.8 | 5.57 | 0 | 23 | 1.2 |
|  | **Irritability (Youth-report)** | | | | |
| ***HV*** | 1.1 | 1.87 | 0 | 10 | 2.1 |
| ***ANX*** | 2.8 | 2.69 | 0 | 10 | 0.9 |
| ***ADHD*** | 2.9 | 2.76 | 0 | 10 | 0.8 |
| ***DMDD*** | 4.6 | 2.7 | 0 | 10 | 0.1 |
|  | **Irritability (Parent-report)** | | | | |
| ***HV*** | 0.8 | 2.52 | 0 | 8 | 2.5 |
| ***ANX*** | 3 | 3.11 | 0 | 10 | 0.3 |
| ***ADHD*** | 3.9 | 3.32 | 0 | 10 | 1 |
| ***DMDD*** | 6.5 | 2.37 | 0 | 10 | -0.8 |
|  | **Anxiety (Youth-report)** | | | | |
| ***HV*** | 10.3 | 12.98 | 0 | 54 | 1.5 |
| ***ANX*** | 29.8 | 12.16 | 2 | 75 | 0.3 |
| ***ADHD*** | 19.6 | 13.03 | 0 | 48 | 0.4 |
| ***DMDD*** | 19.8 | 14.59 | 0 | 58 | 0.5 |
|  | **Anxiety (Parent-report)** | | | | |
| ***HV*** | 14.4 | 12.42 | 0 | 63 | 1.4 |
| ***ANX*** | 18.6 | 13.44 | 2 | 76 | 0.3 |
| ***ADHD*** | 18.6 | 12.94 | 0 | 52 | 0.9 |
| ***DMDD*** | 21.5 | 16.26 | 0 | 71 | 1.2 |
|  | **Depression (Youth-report)** | | | | |
| ***HV*** | 4.3 | 5.2 | 0 | 26 | 1.8 |
| ***ANX*** | 12 | 8.49 | 0 | 44 | 0.9 |
| ***ADHD*** | 8 | 6.75 | 0 | 29 | 1.2 |
| ***DMDD*** | 9.8 | 7.22 | 0 | 29 | 0.9 |

**^Abbreviations: ADHD,^** ^Attention-Deficit/ Hyperactivity Disorder;^ **^ANX,^** ^anxiety disorder;^ **^DMDD,^** ^Disruptive Mood Dysregulation
Disorder;^ **^HV,^** ^Healthy volunteer;^ **^SD,^** ^standard deviation^

**Table S3. Estimates of models 1, 2a and 3a predicting youth-rated anxiety.**

|  | **yANX** |  |  | **yANX** |  |  | **yANX** |  |  | **yANX** |  |  |
| --- | --- | --- | --- | --- | --- | --- | --- | --- | --- | --- | --- | --- |
| *Predictors* | *Estimates* | *CI* | *p* | *Estimates* | *CI* | *p* | *Estimates* | *CI* | *p* | *Estimates* | *CI* | *p* |
| (Intercept) | 18.56 | 17.21 – 19.90 | **<0.001** | 21.02 | 14.59 – 27.46 | **<0.001** | 8.1 | 1.16 – 15.05 | **0.022** | 4.78 | -2.25 – 11.80 | 0.183 |
| Wave |  |  |  | -1.43 | -1.89 – -0.97 | **<0.001** | -1.21 | -2.61 – 0.20 | 0.092 | -0.9 | -2.59 – 0.80 | 0.3 |
| AGE |  |  |  | -0.09 | -0.59 – 0.40 | 0.712 | 0.29 | -0.16 – 0.74 | 0.209 | 0.39 | -0.02 – 0.79 | 0.061 |
| NLE |  |  |  |  |  |  | 1.43 | -0.12 – 2.98 | 0.07 | 0.56 | -1.41 – 2.54 | 0.576 |
| yB-IRR |  |  |  |  |  |  | 1.02 | -0.45 – 2.48 | 0.173 | 1.94 | -0.72 – 4.61 | 0.153 |
| Wave × NLE |  |  |  |  |  |  | 0.12 | -0.47 – 0.72 | 0.691 | -0.11 | -0.88 – 0.66 | 0.775 |
| Wave × yB-IRR |  |  |  |  |  |  | -0.03 | -0.49 – 0.42 | 0.884 | 0.27 | -0.85 – 1.38 | 0.64 |
| NLE × yB-IRR |  |  |  |  |  |  | 0.45 | -0.06 – 0.96 | 0.084 | 0.29 | -0.70 – 1.28 | 0.564 |
| Wave × NLE × yB-IRR |  |  |  |  |  |  | -0.08 | -0.26 – 0.10 | 0.374 | -0.12 | -0.53 – 0.28 | 0.546 |
| ANX |  |  |  |  |  |  |  |  |  | 11.92 | 2.37 – 21.48 | **0.015** |
| ADHD |  |  |  |  |  |  |  |  |  | 7.9 | -4.04 – 19.84 | 0.194 |
| DMDD |  |  |  |  |  |  |  |  |  | 7.29 | -8.10 – 22.68 | 0.353 |
| Wave × ANX |  |  |  |  |  |  |  |  |  | 0.17 | -6.21 – 6.55 | 0.958 |
| Wave × ADHD |  |  |  |  |  |  |  |  |  | -1.56 | -6.86 – 3.74 | 0.563 |
| Wave × DMDD |  |  |  |  |  |  |  |  |  | -1.29 | -7.16 – 4.58 | 0.667 |
| NLE × ANX |  |  |  |  |  |  |  |  |  | 0.71 | -2.79 – 4.20 | 0.691 |
| NLE × ADHD |  |  |  |  |  |  |  |  |  | -2.52 | -7.92 – 2.88 | 0.36 |
| NLE × DMDD |  |  |  |  |  |  |  |  |  | -3.14 | -8.74 – 2.45 | 0.271 |
| yB-IRR × ANX |  |  |  |  |  |  |  |  |  | -1.2 | -5.23 – 2.82 | 0.558 |
| yB-IRR × ADHD |  |  |  |  |  |  |  |  |  | -2.31 | -8.57 – 3.96 | 0.47 |
| yB-IRR × DMDD |  |  |  |  |  |  |  |  |  | -2.86 | -6.76 – 1.03 | 0.149 |
| Wave × NLE × ANX |  |  |  |  |  |  |  |  |  | 0.1 | -2.02 – 2.22 | 0.927 |
| Wave × NLE × ADHD |  |  |  |  |  |  |  |  |  | 1.16 | -1.19 – 3.51 | 0.333 |
| Wave × NLE × DMDD |  |  |  |  |  |  |  |  |  | 1.2 | -0.91 – 3.31 | 0.264 |
| Wave × yB-IRR × ANX |  |  |  |  |  |  |  |  |  | -2.9 | -5.35 – -0.45 | **0.02** |
| Wave × yB-IRR × ADHD |  |  |  |  |  |  |  |  |  | 1.25 | -2.12 – 4.61 | 0.468 |
| Wave × yB-IRR × DMDD |  |  |  |  |  |  |  |  |  | 0.08 | -1.40 – 1.56 | 0.915 |
| NLE × yB-IRR × ANX |  |  |  |  |  |  |  |  |  | 0.07 | -1.31 – 1.44 | 0.923 |
| NLE × yB-IRR × ADHD |  |  |  |  |  |  |  |  |  | 1.37 | -1.23 – 3.96 | 0.301 |
| NLE × yB-IRR × DMDD |  |  |  |  |  |  |  |  |  | 0.81 | -0.60 – 2.22 | 0.259 |
| Wave × NLE × yB-IRR × ANX |  |  |  |  |  |  |  |  |  | 0.88 | 0.10 – 1.67 | **0.028** |
| Wave × NLE × yB-IRR × ADHD |  |  |  |  |  |  |  |  |  | -0.7 | -1.89 – 0.49 | 0.25 |
| Wave × NLE × yB-IRR × DMDD |  |  |  |  |  |  |  |  |  | -0.2 | -0.74 – 0.35 | 0.48 |
| **Random Effects** |  |  |  |  |  |  |  |  |  |  |  |  |
| σ^2^ | 55.17 |  |  | 52.73 |  |  | 52.37 |  |  | 51.9 |  |  |
| τ_00_ | 151.84 |  |  | 149.21 |  |  | 113.33 |  |  | 84.79 |  |  |
| ICC | 0.73 |  |  | 0.74 |  |  | 0.68 |  |  | 0.62 |  |  |
| N | 409 |  |  | 409 |  |  | 409 |  |  | 409 |  |  |
| Observations | 1063 |  |  | 1063 |  |  | 1063 |  |  | 1063 |  |  |
| Marginal R^2^ / Conditional R^2^ | 0 | / 0.733 |  | 0.013 | / 0.742 |  | 0.191 | / 0.744 |  | 0.343 | / 0.751 |  |

**Abbreviations:** NLE: Negative Life Events; pBIRR: Baseline Parent-Rated Irritability; yBIRR: Baseline Youth-Rated Irritability; CI: Confidence Interval; ICC: Intraclass Correlation Coeffecient; N: Number of Participants

**Table S4. Comparison between models 1, 2a, 2b, 3a and 3b predicting youth-rated anxiety.**

| **Model** |  | **npar** | **AIC** | **BIC** | **logLik** | **deviance** | **Chisq** | **Df** | **P value** |
| --- | --- | --- | --- | --- | --- | --- | --- | --- | --- |
|  | **Constant** | 3 | 7362.99 | 7377.6 | -3678.49 | 7356.99 |  |  |  |
| **1** | **Wave** | 5 | 7330.29 | 7354.64 | -3660.15 | 7320.29 | 36.69 | 2 | <.001 |
| **2a** | **Wave*NLE*yBIRR** | 11 | 7244.86 | 7298.43 | -3611.43 | 7222.86 | 97.43 | 6 | <.001 |
| **2b** | **Wave*NLE*pBIRR** | 11 | 7299.80 | 7353.37 | -3638.90 | 7277.80 |  |  |  |
| **3a** | **Wave*NLE*yBIRR*SC** | 35 | 7174.46 | 7344.91 | -3552.23 | 7104.46 | 173.34 | 24 | <.001 |
| **3b** | **Wave*NLE*pBIRR*SC** | 35 | 7227.92 | 7398.38 | -3578.96 | 7157.92 |  |  |  |
| **Abbreviations:** NLE: Negative Life Events; pBIRR: Baseline Parent-Rated Irritability; yBIRR: Baseline Youth-Rated Irritability; AIC: Akaike Information Criterion; BIC: Bayesian Information Criterion; npar: number of parameters; logLik: Log-Likelihood; Df: Degrees of Freedom | | | | | | | | | |

**Table S5. Estimates of models 1, 2a and 3a predicting parent-rated anxiety.**

|  | **pANX** |  |  | **pANX** |  |  | **pANX** |  |  | **pANX** |  |  |
| --- | --- | --- | --- | --- | --- | --- | --- | --- | --- | --- | --- | --- |
| *Predictors* | *Estimates* | *CI* | *p* | *Estimates* | *CI* | *p* | *Estimates* | *CI* | *p* | *Estimates* | *CI* | *p* |
| (Intercept) | 17.04 | 15.66 – 18.41 | **<0.001** | 28.12 | 21.62 – 34.63 | **<0.001** | 15.98 | 8.76 – 23.20 | **<0.001** | 13 | 6.35 – 19.65 | **<0.001** |
| Wave |  |  |  | -0.92 | -1.34 – -0.49 | **<0.001** | -1.02 | -2.25 – 0.20 | 0.102 | -0.46 | -1.80 – 0.88 | 0.501 |
| AGE |  |  |  | -0.81 | -1.31 – -0.31 | **0.002** | -0.63 | -1.09 – -0.16 | **0.009** | -0.53 | -0.92 – -0.13 | **0.009** |
| NLE |  |  |  |  |  |  | 2.68 | 1.13 – 4.22 | **0.001** | 0.54 | -1.27 – 2.35 | 0.558 |
| pBIRR |  |  |  |  |  |  | 2.41 | 1.21 – 3.61 | **<0.001** | 3.15 | 0.70 – 5.60 | **0.012** |
| Wave × NLE |  |  |  |  |  |  | 0.2 | -0.31 – 0.70 | 0.439 | 0.09 | -0.50 – 0.68 | 0.758 |
| Wave × pBIRR |  |  |  |  |  |  | -0.09 | -0.41 – 0.22 | 0.56 | 0.65 | -0.52 – 1.82 | 0.277 |
| NLE × pBIRR |  |  |  |  |  |  | -0.33 | -0.76 – 0.09 | 0.124 | 0.2 | -0.75 – 1.16 | 0.677 |
| Wave × NLE × pBIRR |  |  |  |  |  |  | -0.02 | -0.14 – 0.10 | 0.713 | -0.33 | -0.80 – 0.15 | 0.175 |
| ANX |  |  |  |  |  |  |  |  |  | 22.87 | 13.90 – 31.84 | **<0.001** |
| ADHD |  |  |  |  |  |  |  |  |  | 5.93 | -4.81 – 16.66 | 0.279 |
| DMDD |  |  |  |  |  |  |  |  |  | 16.43 | -13.10 – 45.95 | 0.275 |
| Wave × ANX |  |  |  |  |  |  |  |  |  | -5.58 | -11.36 – 0.20 | 0.059 |
| Wave × ADHD |  |  |  |  |  |  |  |  |  | 0.59 | -3.89 – 5.07 | 0.797 |
| Wave × DMDD |  |  |  |  |  |  |  |  |  | -4.65 | -14.26 – 4.97 | 0.343 |
| NLE × ANX |  |  |  |  |  |  |  |  |  | -1.15 | -4.40 – 2.09 | 0.486 |
| NLE × ADHD |  |  |  |  |  |  |  |  |  | 1.38 | -3.06 – 5.82 | 0.542 |
| NLE × DMDD |  |  |  |  |  |  |  |  |  | -2 | -11.91 – 7.92 | 0.693 |
| pBIRR × ANX |  |  |  |  |  |  |  |  |  | -2.11 | -5.45 – 1.22 | 0.214 |
| pBIRR × ADHD |  |  |  |  |  |  |  |  |  | -0.25 | -4.27 – 3.76 | 0.901 |
| pBIRR × DMDD |  |  |  |  |  |  |  |  |  | -4.04 | -9.02 – 0.94 | 0.112 |
| Wave × NLE × ANX |  |  |  |  |  |  |  |  |  | 1.88 | 0.02 – 3.75 | **0.048** |
| Wave × NLE × ADHD |  |  |  |  |  |  |  |  |  | -0.79 | -3.05 – 1.47 | 0.494 |
| Wave × NLE × DMDD |  |  |  |  |  |  |  |  |  | 0.74 | -2.21 – 3.69 | 0.625 |
| Wave × pBIRR × ANX |  |  |  |  |  |  |  |  |  | -2.96 | -4.82 – -1.11 | **0.002** |
| Wave × pBIRR × ADHD |  |  |  |  |  |  |  |  |  | -2.18 | -3.99 – -0.38 | **0.018** |
| Wave × pBIRR × DMDD |  |  |  |  |  |  |  |  |  | 0.09 | -1.65 – 1.83 | 0.921 |
| NLE × pBIRR × ANX |  |  |  |  |  |  |  |  |  | -0.36 | -1.57 – 0.85 | 0.56 |
| NLE × pBIRR × ADHD |  |  |  |  |  |  |  |  |  | -1.1 | -2.71 – 0.51 | 0.18 |
| NLE × pBIRR × DMDD |  |  |  |  |  |  |  |  |  | 0.25 | -1.47 – 1.96 | 0.779 |
| Wave × NLE × pBIRR × ANX |  |  |  |  |  |  |  |  |  | 0.86 | 0.22 – 1.50 | **0.009** |
| Wave × NLE × pBIRR × ADHD |  |  |  |  |  |  |  |  |  | 0.93 | 0.16 – 1.70 | **0.018** |
| Wave × NLE × pBIRR × DMDD |  |  |  |  |  |  |  |  |  | 0.14 | -0.48 – 0.76 | 0.658 |
| **Random Effects** |  |  |  |  |  |  |  |  |  |  |  |  |
| σ^2^ | 44.23 |  |  | 43.42 |  |  | 43.27 |  |  | 39.97 |  |  |
| τ_00_ | 159.65 |  |  | 152.76 |  |  | 128.07 |  |  | 85.54 |  |  |
| ICC | 0.78 |  |  | 0.78 |  |  | 0.75 |  |  | 0.68 |  |  |
| N | 423 |  |  | 423 |  |  | 423 |  |  | 423 |  |  |
| Observations | 1143 |  |  | 1143 |  |  | 1143 |  |  | 1143 |  |  |
| Marginal R^2^ / Conditional R^2^ | 0 | / 0.783 |  | 0.028 | / 0.785 |  | 0.162 | / 0.788 |  | 0.389 | / 0.805 |  |

**Abbreviations:** NLE: Negative Life Events; pBIRR: Baseline Parent-Rated Irritability; yBIRR: Baseline Youth-Rated Irritability; CI: Confidence Interval; ICC: Intraclass Correlation Coeffecient; N: Number of Participants; ID: Participant

**Table S6. Comparison between models 1, 2a, 2b, 3a, and 3b all predicting parent-rated anxiety.**

| **Model** |  | **npar** | **AIC** | **BIC** | **logLik** | **deviance** | **Chisq** | **Df** | **P value** |
| --- | --- | --- | --- | --- | --- | --- | --- | --- | --- |
|  | **Constant** | 3 | 7090.93 | 7105.48 | -3542.46 | 7084.93 |  |  |  |
| **1** | **Wave** | 5 | 7067.09 | 7091.33 | -3528.54 | 7057.09 | 27.84 | 2 | <.001 |
| **2a** | **Wave*NLE*pBIRR** | 11 | 7014.24 | 7067.58 | -3496.12 | 6992.24 | 64.85 | 6 | <.001 |
| **2b** | **Wave*NLE*yBIRR** | 11 | 7016.69 | 7070.03 | -3497.34 | 6994.69 |  |  |  |
| **3a** | **Wave*NLE*pBIRR*SC** | 35 | 6861.78 | 7031.50 | -3395.89 | 6791.78 | 202.90 | 24 | <.001 |
| **3b** | **Wave*NLE*yBIRR*SC** | 35 | 6913.21 | 7082.93 | -3421.61 | 6843.21 |  |  |  |
| **Abbreviations:** NLE: Negative Life Events; pBIRR: Baseline Parent-Rated Irritability; yBIRR: Baseline Youth-Rated Irritability; SC: Syndromal Context; AIC: Akaike Information Criterion; BIC: Bayesian Information Criterion; npar: Non-Parametric; logLik: Log-Likelihood; Df: Degrees of Freedom | | | | | | | | | |


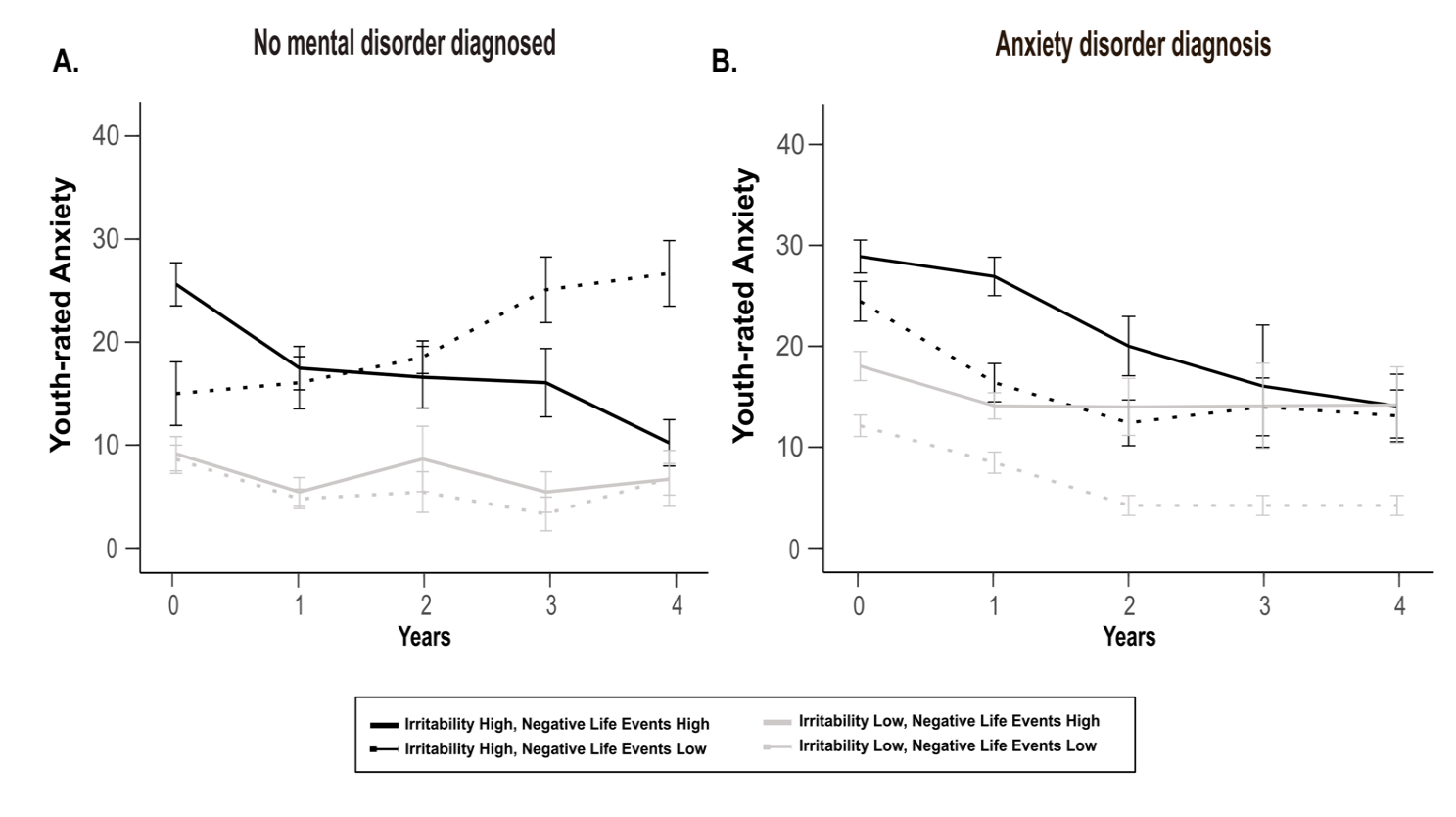


**Figure S1. Trajectories of youth-rated anxiety in the absence of a psychiatric disorder diagnosis and in youth diagnosed with an anxiety disorder.**

**
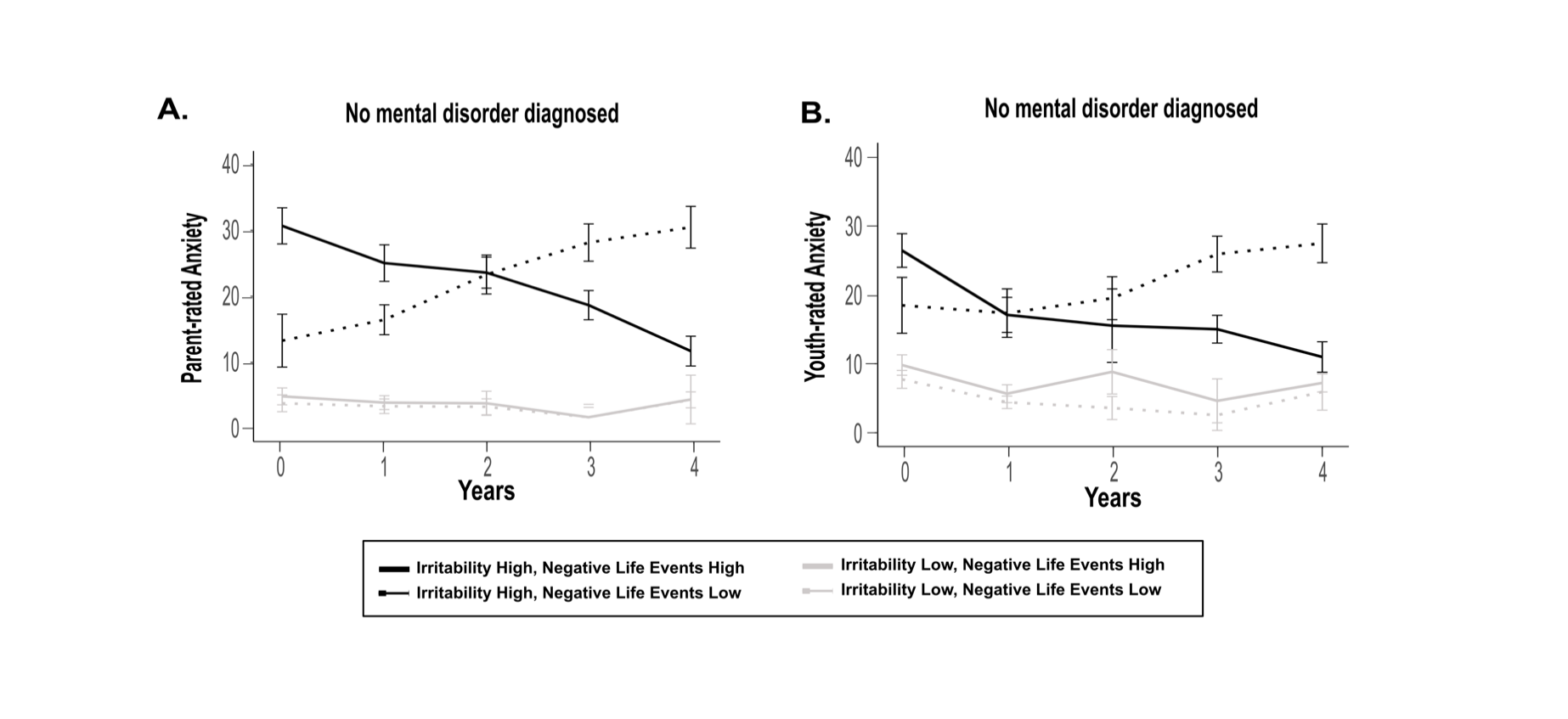
**

**Figure S2. Trajectories of parent- and youth-rated anxiety in the absence of a psychiatric disorder diagnosis using a cut-off value of >= 2 on the Affective Reactivity Index to define “High Irritability” in this group.** Size of the subgroups is: high irritability, high NLE, n = 23; high irritability, low NLE, n_HV_ = 27; low irritability, high NLE, n_HV_ = 81; low irritability, low NLE, n_HV_ = 78).

| **Table S7. Estimates of models 1, 2a and 3a predicting depression.**   \|  \| **DEP** \|  \|  \| **DEP** \|  \|  \| **DEP** \|  \|  \| **DEP** \|  \|  \| \| --- \| --- \| --- \| --- \| --- \| --- \| --- \| --- \| --- \| --- \| --- \| --- \| --- \| \| *Predictors* \| *Estimates* \| *CI* \| *p* \| *Estimates* \| *CI* \| *p* \| *Estimates* \| *CI* \| *p* \| *Estimates* \| *CI* \| *p* \| \| (Intercept) \| 6.68 \| 5.88 – 7.48 \| **<0.001** \| 6.43 \| 2.46 – 10.39 \| **0.002** \| -0.83 \| -4.67 – 3.00 \| 0.67 \| -2.37 \| -6.51 – 1.77 \| 0.262 \| \| Wave \|  \|  \|  \| -0.24 \| -0.56 – 0.07 \| 0.132 \| 0 \| -1.01 – 1.01 \| 0.998 \| -0.07 \| -1.26 – 1.11 \| 0.902 \| \| AGE \|  \|  \|  \| 0.04 \| -0.27 – 0.34 \| 0.806 \| 0.28 \| 0.03 – 0.53 \| **0.026** \| 0.34 \| 0.10 – 0.58 \| **0.006** \| \| NLE \|  \|  \|  \|  \|  \|  \| 0.5 \| -0.39 – 1.40 \| 0.267 \| 0.26 \| -0.87 – 1.40 \| 0.649 \| \| yBIRR \|  \|  \|  \|  \|  \|  \| 0.75 \| -0.04 – 1.54 \| 0.061 \| 0.59 \| -1.06 – 2.25 \| 0.482 \| \| Wave × NLE \|  \|  \|  \|  \|  \|  \| -0.03 \| -0.46 – 0.41 \| 0.901 \| 0.03 \| -0.50 – 0.56 \| 0.913 \| \| Wave × yBIRR \|  \|  \|  \|  \|  \|  \| -0.26 \| -0.56 – 0.03 \| 0.079 \| 0.24 \| -0.52 – 0.99 \| 0.536 \| \| NLE × yBIRR \|  \|  \|  \|  \|  \|  \| 0.24 \| -0.04 – 0.51 \| 0.091 \| 0.36 \| -0.24 – 0.95 \| 0.24 \| \| Wave × NLE × yBIRR \|  \|  \|  \|  \|  \|  \| 0.07 \| -0.05 – 0.18 \| 0.269 \| -0.14 \| -0.42 – 0.13 \| 0.296 \| \| ANX \|  \|  \|  \|  \|  \|  \|  \|  \|  \| 12.17 \| 4.55 – 19.78 \| **0.002** \| \| ADHD \|  \|  \|  \|  \|  \|  \|  \|  \|  \| 0.09 \| -6.52 – 6.70 \| 0.979 \| \| DMDD \|  \|  \|  \|  \|  \|  \|  \|  \|  \| 3.68 \| -3.98 – 11.35 \| 0.346 \| \| Wave × ANX \|  \|  \|  \|  \|  \|  \|  \|  \|  \| -10.2 \| -16.95 – -3.46 \| **0.003** \| \| Wave × ADHD \|  \|  \|  \|  \|  \|  \|  \|  \|  \| -0.91 \| -4.25 – 2.43 \| 0.593 \| \| Wave × DMDD \|  \|  \|  \|  \|  \|  \|  \|  \|  \| 1.78 \| -1.72 – 5.29 \| 0.318 \| \| NLE × ANX \|  \|  \|  \|  \|  \|  \|  \|  \|  \| -2.09 \| -4.81 – 0.63 \| 0.132 \| \| NLE × ADHD \|  \|  \|  \|  \|  \|  \|  \|  \|  \| 0.52 \| -2.39 – 3.43 \| 0.726 \| \| NLE × DMDD \|  \|  \|  \|  \|  \|  \|  \|  \|  \| -0.4 \| -3.21 – 2.41 \| 0.779 \| \| yBIRR × ANX \|  \|  \|  \|  \|  \|  \|  \|  \|  \| -2.85 \| -6.14 – 0.44 \| 0.09 \| \| yBIRR × ADHD \|  \|  \|  \|  \|  \|  \|  \|  \|  \| -0.08 \| -4.02 – 3.87 \| 0.97 \| \| yBIRR × DMDD \|  \|  \|  \|  \|  \|  \|  \|  \|  \| -0.24 \| -2.40 – 1.92 \| 0.825 \| \| Wave × NLE × ANX \|  \|  \|  \|  \|  \|  \|  \|  \|  \| 2.96 \| 0.59 – 5.34 \| **0.015** \| \| Wave × NLE × ADHD \|  \|  \|  \|  \|  \|  \|  \|  \|  \| 0.58 \| -0.86 – 2.03 \| 0.429 \| \| Wave × NLE × DMDD \|  \|  \|  \|  \|  \|  \|  \|  \|  \| -0.73 \| -2.04 – 0.59 \| 0.279 \| \| Wave × yBIRR × ANX \|  \|  \|  \|  \|  \|  \|  \|  \|  \| 2.75 \| 0.39 – 5.11 \| **0.023** \| \| Wave × yBIRR × ADHD \|  \|  \|  \|  \|  \|  \|  \|  \|  \| -0.33 \| -2.46 – 1.79 \| 0.757 \| \| Wave × yBIRR × DMDD \|  \|  \|  \|  \|  \|  \|  \|  \|  \| -0.8 \| -1.75 – 0.15 \| 0.098 \| \| NLE × yBIRR × ANX \|  \|  \|  \|  \|  \|  \|  \|  \|  \| 0.72 \| -0.31 – 1.75 \| 0.169 \| \| NLE × yBIRR × ADHD \|  \|  \|  \|  \|  \|  \|  \|  \|  \| 0.03 \| -1.33 – 1.40 \| 0.96 \| \| NLE × yBIRR × DMDD \|  \|  \|  \|  \|  \|  \|  \|  \|  \| -0.13 \| -0.90 – 0.65 \| 0.75 \| \| Wave × NLE × yBIRR × ANX \|  \|  \|  \|  \|  \|  \|  \|  \|  \| -0.67 \| -1.38 – 0.04 \| 0.064 \| \| Wave × NLE × yBIRR × ADHD \|  \|  \|  \|  \|  \|  \|  \|  \|  \| 0.2 \| -0.47 – 0.88 \| 0.558 \| \| Wave × NLE × yBIRR × DMDD \|  \|  \|  \|  \|  \|  \|  \|  \|  \| 0.33 \| -0.02 – 0.68 \| 0.066 \| \| **Random Effects** \|  \|  \|  \|  \|  \|  \|  \|  \|  \|  \|  \|  \| \| σ^2^ \| 17.73 \|  \|  \| 17.7 \|  \|  \| 17.7 \|  \|  \| 17.21 \|  \|  \| \| τ_00_ \| 34.21 \|  \|  \| 34.29 \|  \|  \| 19.06 \|  \|  \| 16.61 \|  \|  \| \| ICC \| 0.66 \|  \|  \| 0.66 \|  \|  \| 0.52 \|  \|  \| 0.49 \|  \|  \| \| N \| 347 \|  \|  \| 347 \|  \|  \| 347 \|  \|  \| 347 \|  \|  \| \| Observations \| 759 \|  \|  \| 759 \|  \|  \| 759 \|  \|  \| 759 \|  \|  \| \| Marginal R^2^ / Conditional R^2^ \| 0 \| / 0.659 \|  \| 0.002 \| / 0.660 \|  \| 0.296 \| / 0.661 \|  \| 0.367 \| / 0.678 \|  \|   **Abbreviations:** NLE: Negative Life Events; pBIRR: Baseline Parent-Rated Irritability; yBIRR: Baseline Youth-Rated Irritability; CI: Confidence Interval; ICC: Intraclass Correlation Coeffecient; N: Number of Participants |
| --- | --- | --- | --- | --- | --- | --- | --- | --- | --- | --- | --- | --- | --- | --- | --- | --- | --- | --- | --- | --- | --- | --- | --- | --- | --- | --- | --- | --- | --- | --- | --- | --- | --- | --- | --- | --- | --- | --- | --- | --- | --- | --- | --- | --- | --- | --- | --- | --- | --- | --- | --- | --- | --- | --- | --- | --- | --- | --- | --- | --- | --- | --- | --- | --- | --- | --- | --- | --- | --- | --- | --- | --- | --- | --- | --- | --- | --- | --- | --- | --- | --- | --- | --- | --- | --- | --- | --- | --- | --- | --- | --- | --- | --- | --- | --- | --- | --- | --- | --- | --- | --- | --- | --- | --- | --- | --- | --- | --- | --- | --- | --- | --- | --- | --- | --- | --- | --- | --- | --- | --- | --- | --- | --- | --- | --- | --- | --- | --- | --- | --- | --- | --- | --- | --- | --- | --- | --- | --- | --- | --- | --- | --- | --- | --- | --- | --- | --- | --- | --- | --- | --- | --- | --- | --- | --- | --- | --- | --- | --- | --- | --- | --- | --- | --- | --- | --- | --- | --- | --- | --- | --- | --- | --- | --- | --- | --- | --- | --- | --- | --- | --- | --- | --- | --- | --- | --- | --- | --- | --- | --- | --- | --- | --- | --- | --- | --- | --- | --- | --- | --- | --- | --- | --- | --- | --- | --- | --- | --- | --- | --- | --- | --- | --- | --- | --- | --- | --- | --- | --- | --- | --- | --- | --- | --- | --- | --- | --- | --- | --- | --- | --- | --- | --- | --- | --- | --- | --- | --- | --- | --- | --- | --- | --- | --- | --- | --- | --- | --- | --- | --- | --- | --- | --- | --- | --- | --- | --- | --- | --- | --- | --- | --- | --- | --- | --- | --- | --- | --- | --- | --- | --- | --- | --- | --- | --- | --- | --- | --- | --- | --- | --- | --- | --- | --- | --- | --- | --- | --- | --- | --- | --- | --- | --- | --- | --- | --- | --- | --- | --- | --- | --- | --- | --- | --- | --- | --- | --- | --- | --- | --- | --- | --- | --- | --- | --- | --- | --- | --- | --- | --- | --- | --- | --- | --- | --- | --- | --- | --- | --- | --- | --- | --- | --- | --- | --- | --- | --- | --- | --- | --- | --- | --- | --- | --- | --- | --- | --- | --- | --- | --- | --- | --- | --- | --- | --- | --- | --- | --- | --- | --- | --- | --- | --- | --- | --- | --- | --- | --- | --- | --- | --- | --- | --- | --- | --- | --- | --- | --- | --- | --- | --- | --- | --- | --- | --- | --- | --- | --- | --- | --- | --- | --- | --- | --- | --- | --- | --- | --- | --- | --- | --- | --- | --- | --- | --- | --- | --- | --- | --- | --- | --- | --- | --- | --- | --- | --- | --- | --- | --- | --- | --- | --- | --- | --- | --- | --- | --- | --- | --- | --- | --- | --- | --- | --- | --- | --- | --- | --- | --- | --- | --- | --- | --- | --- | --- | --- | --- | --- | --- | --- | --- | --- | --- | --- | --- | --- | --- | --- | --- | --- | --- | --- | --- | --- | --- | --- | --- | --- | --- | --- | --- | --- | --- | --- | --- | --- | --- | --- | --- | --- | --- | --- | --- | --- | --- | --- | --- | --- | --- | --- | --- | --- | --- | --- | --- | --- | --- | --- | --- | --- | --- | --- | --- | --- | --- | --- | --- | --- | --- | --- | --- | --- | --- | --- | --- | --- | --- | --- | --- | --- | --- | --- | --- | --- | --- | --- | --- | --- | --- | --- | --- | --- | --- | --- | --- | --- | --- | --- | --- | --- | --- | --- | --- | --- | --- | --- |

**Table S8. Comparison between models 1, 3a, 2b, 3a, and 3b all predicting depression.**

| **Model** |  | **npar** | **AIC** | **BIC** | **logLik** | **deviance** | **Chisq** | **Df** | **P value** |
| --- | --- | --- | --- | --- | --- | --- | --- | --- | --- |
|  | **Constant** | 3 | 4211.16 | 4224.63 | -2102.58 | 4205.16 |  |  |  |
| **1** | **Wave** | 5 | 4212.80 | 4235.26 | -2101.40 | 4202.80 | 2.35 | 2 | 0.308 |
| **2a** | **Wave*NLE*pBIRR** | 11 | 4171.46 | 4220.85 | -2074.73 | 4149.46 | 53.35 | 6 | <.001 |
| **2b** | **Wave*NLE*yBIRR** | 11 | 4105.41 | 4154.80 | -2041.70 | 4083.41 | 10.05 |  |  |
| **3a** | **Wave*NLE*pBIRR*SC** | 35 | 4042.91 | 4100.09 | -2036.46 | 4072.91 | 73.49 | 24 | <.001 |
| **3b** | **Wave*NLE*yBIRR*SC** | 35 | 4090.47 | 4147.64 | -2010.23 | 4020.47 | 52.45 |  |  |
| **Abbreviations:** NLE: Negative Life Events; pBIRR: Baseline Parent-Rated Irritability; yBIRR: Baseline Youth-Rated Irritability; SC: Syndromal Context; AIC: Akaike Information Criterion; BIC: Bayesian Information Criterion; npar: number of parameters; logLik: Log-Likelihood; Df: Degrees of Freedom | | | | | | | | | |

**Table S9. Estimates of models 1, 2c and 3c predicting youth-rated irritability.**

|  | **yIRR** |  |  | **yIRR** |  |  | **yIRR** |  |  | **yIRR** |  |  |
| --- | --- | --- | --- | --- | --- | --- | --- | --- | --- | --- | --- | --- |
| *Predictors* | *Estimates* | *CI* | *p* | *Estimates* | *CI* | *p* | *Estimates* | *CI* | *p* | *Estimates* | *CI* | *p* |
| (Intercept) | 1.75 | 1.53 – 1.97 | **<0.001** | 3.28 | 2.17 – 4.39 | **<0.001** | 2.13 | 0.97 – 3.30 | **<0.001** | 2 | 0.83 – 3.18 | **0.001** |
| Wave |  |  |  | -0.29 | -0.38 – -0.21 | **<0.001** | -0.14 | -0.34 – 0.07 | 0.194 | -0.09 | -0.39 – 0.21 | 0.545 |
| AGE |  |  |  | -0.09 | -0.18 – -0.01 | **0.025** | -0.11 | -0.19 – -0.03 | **0.007** | -0.11 | -0.18 – -0.04 | **0.003** |
| NLE |  |  |  |  |  |  | 0.56 | 0.34 – 0.79 | **<0.001** | 0.32 | -0.11 – 0.53 | **0.002** |
| Wave × NLE |  |  |  |  |  |  | -0.06 | -0.14 – 0.01 | 0.104 | -0.01 | -0.14 – 0.11 | 0.837 |
| ANX |  |  |  |  |  |  |  |  |  | 0.42 | -1.11 – 1.95 | 0.593 |
| ADHD |  |  |  |  |  |  |  |  |  | -0.93 | -2.83 – 0.97 | 0.336 |
| DMDD |  |  |  |  |  |  |  |  |  | 3.16 | 1.78 – 4.55 | **<0.001** |
| Wave × ANX |  |  |  |  |  |  |  |  |  | -0.13 | -1.20 – 0.94 | 0.806 |
| Wave × ADHD |  |  |  |  |  |  |  |  |  | -0.3 | -1.08 – 0.48 | 0.447 |
| Wave × DMDD |  |  |  |  |  |  |  |  |  | -0.49 | -0.67 – -0.31 | **<0.001** |
| NLE × ANX |  |  |  |  |  |  |  |  |  | 0.32 | -0.23 – 0.87 | 0.249 |
| NLE × ADHD |  |  |  |  |  |  |  |  |  | 0.46 | -0.31 – 1.24 | 0.242 |
| NLE × DMDD |  |  |  |  |  |  |  |  |  | -0.02 | -0.53 – 0.49 | 0.931 |
| Wave × NLE × ANX |  |  |  |  |  |  |  |  |  | 0.04 | -0.33 – 0.40 | 0.847 |
| Wave × NLE × ADHD |  |  |  |  |  |  |  |  |  | 0.2 | -0.10 – 0.51 | 0.194 |
| Wave × NLE × DMDD |  |  |  |  |  |  |  |  |  | -0.06 | -0.22 – 0.11 | 0.515 |
| **Random Effects** |  |  |  |  |  |  |  |  |  |  |  |  |
| σ^2^ | 2.04 |  |  | 1.85 |  |  | 1.85 |  |  | 1.75 |  |  |
| τ_00_ | 3.17 |  |  | 3.3 |  |  | 3.03 |  |  | 2.19 |  |  |
| ICC | 0.61 |  |  | 0.64 |  |  | 0.62 |  |  | 0.56 |  |  |
| N | 427 |  |  | 427 |  |  | 427 |  |  | 427 |  |  |
| Observations | 1467 |  |  | 1467 |  |  | 1467 |  |  | 1467 |  |  |
| Marginal R^2^ / Conditional R^2^ | 0 | / 0.609 |  | 0.036 | / 0.653 |  | 0.087 | / 0.654 |  | 0.267 | / 0.675 |  |

**Abbreviations:** NLE: Negative Life Events; pBIRR: Baseline Parent-Rated Irritability; yBIRR: Baseline Youth-Rated Irritability; CI: Confidence Interval; ICC: Intraclass Correlation Coeffecient; N: Number of Participants

|  |  |
| --- | --- |

**Table S10. Comparison between models 1, 2c and 3c predicting youth-rated irritability.**

| **Model** |  | **npar** | **AIC** | **BIC** | **logLik** | **deviance** | **Chisq** | **Df** | **P value** |
| --- | --- | --- | --- | --- | --- | --- | --- | --- | --- |
|  | **Constant** | 3 | 3529.35 | 3543.59 | -1761.68 | 3523.35 |  |  |  |
| **1** | **Wave** | 5 | 3483.96 | 3507.69 | -1736.98 | 3473.96 | 49.39 | 2 | <.001 |
| **2c** | **Wave*NLE** | 7 | 3463.57 | 3496.79 | -1724.78 | 3449.57 | 24.39 | 2 | <.001 |
| **3c** | **Wave*NLE*SC** | 19 | 3362.06 | 3452.24 | -1662.03 | 3324.06 | 125.50 | 12 | <.001 |

**Abbreviations:** NLE: Negative Life Events; pBIRR: Baseline Parent-Rated Irritability; yBIRR: Baseline Youth-Rated Irritability; SC: Syndromal Context; AIC: Akaike Information Criterion; BIC: Bayesian Information Criterion; npar: number of parameters; logLik: Log-Likelihood; Df: Degrees of Freedom

**Table S11. Estimates of models 1, 2c and 3c predicting parent-rated irritability.**

|  | **pIRR** | | | **pIRR** | | | **pIRR** | | | **pIRR** | | |
| --- | --- | --- | --- | --- | --- | --- | --- | --- | --- | --- | --- | --- |
| *Predictors* | *Estimates* | *CI* | *p* | *Estimates* | *CI* | *p* | *Estimates* | *CI* | *p* | *Estimates* | *CI* | *p* |
| (Intercept) | 2.13 | 1.83 – 2.42 | **<0.001** | 3.92 | 2.45 – 5.40 | **<0.001** | 2.68 | 1.12 – 4.24 | **0.001** | 2.29 | 1.00 – 3.57 | **0.001** |
| Wave |  |  |  | -0.19 | -0.27 – -0.10 | **<0.001** | -0.09 | -0.30 – 0.11 | 0.378 | -0.01 | -0.32 – 0.30 | 0.938 |
| AGE |  |  |  | -0.12 | -0.23 – -0.01 | **0.029** | -0.14 | -0.24 – -0.03 | **0.014** | -0.14 | -0.22 – -0.06 | **<0.001** |
| NLE |  |  |  |  |  |  | 0.59 | 0.31 – 0.87 | **<0.001** | 0.14 | -0.20 – 0.47 | 0.421 |
| Wave × NLE |  |  |  |  |  |  | -0.04 | -0.12 – 0.04 | 0.327 | 0 | -0.13 – 0.13 | 0.969 |
| ANX |  |  |  |  |  |  |  |  |  | 1.05 | -0.64 – 2.73 | 0.224 |
| ADHD |  |  |  |  |  |  |  |  |  | 0.95 | -0.91 – 2.82 | 0.315 |
| DMDD |  |  |  |  |  |  |  |  |  | 5.31 | 3.84 – 6.78 | **<0.001** |
| Wave × ANX |  |  |  |  |  |  |  |  |  | -0.22 | -1.30 – 0.86 | 0.695 |
| Wave × ADHD |  |  |  |  |  |  |  |  |  | -0.09 | -0.86 – 0.67 | 0.815 |
| Wave × DMDD |  |  |  |  |  |  |  |  |  | -0.45 | -0.89 – -0.01 | **0.044** |
| NLE × ANX |  |  |  |  |  |  |  |  |  | 0.17 | -0.44 – 0.77 | 0.594 |
| NLE × ADHD |  |  |  |  |  |  |  |  |  | 0.06 | -0.70 – 0.83 | 0.868 |
| NLE × DMDD |  |  |  |  |  |  |  |  |  | 0.02 | -0.52 – 0.57 | 0.93 |
| Wave × NLE × ANX |  |  |  |  |  |  |  |  |  | -0.03 | -0.39 – 0.33 | 0.852 |
| Wave × NLE × ADHD |  |  |  |  |  |  |  |  |  | 0.09 | -0.21 – 0.39 | 0.547 |
| Wave × NLE × DMDD |  |  |  |  |  |  |  |  |  | -0.01 | -0.18 – 0.16 | 0.939 |
| **Random Effects** |  |  |  |  |  |  |  |  |  |  |  |  |
| σ^2^ | 1.89 |  |  | 1.81 |  |  | 1.81 |  |  | 1.73 |  |  |
| τ_00_ | 6.02 |  |  | 6.14 |  |  | 5.82 |  |  | 2.65 |  |  |
| ICC | 0.76 |  |  | 0.77 |  |  | 0.76 |  |  | 0.61 |  |  |
| N | 432 |  |  | 432 |  |  | 432 |  |  | 432 |  |  |
| Observations | 1147 |  |  | 1147 |  |  | 1147 |  |  | 1147 |  |  |
| Marginal R^2^ / Conditional R^2^ | 0 | / 0.761 |  | 0.019 | / 0.777 |  | 0.061 | / 0.777 |  | 0.483 | / 0.796 |  |

**Abbreviations:** NLE: Negative Life Events; pBIRR: Baseline Parent-Rated Irritability; yBIRR: Baseline Youth-Rated Irritability; CI: Confidence Interval; ICC: Intraclass Correlation Coeffecient; N: Number of Participants

**Table S12. Comparison between models 1, 2c and 3c predicting parent-rated irritability.**

| **Model** |  | **npar** | **AIC** | **BIC** | **logLik** | **deviance** | **Chisq** | **Df** | **P value** |
| --- | --- | --- | --- | --- | --- | --- | --- | --- | --- |
|  | **Constant** | 3 | 3604.11 | 3618.31 | -1799.05 | 3598.11 |  |  |  |
| **1** | **Wave** | 5 | 3584.93 | 3608.59 | -1787.46 | 3574.93 | 23.18 | 2 | <.001 |
| **2c** | **Wave*NLE** | 7 | 3572.50 | 3605.64 | -1779.25 | 3558.50 | 16.42 | 2 | <.001 |
| **3c** | **Wave*NLE*SC** | 19 | 3358.00 | 3447.93 | -1660.00 | 3320.00 | 238.51 | 12 | <.001 |

**Abbreviations:** NLE: Negative Life Events; pBIRR: Baseline Parent-Rated Irritability; yBIRR: Baseline Youth-Rated Irritability; SC: Syndromal Context; AIC: Akaike Information Criterion; BIC: Bayesian Information Criterion; npar: Number of Parameters; logLik: Log-Likelihood; Df: Degrees of Freedom
